# Activation dynamics study of trapped platelets using a lab-built optical tweezers micro-Raman spectrometer

**DOI:** 10.1038/s41598-025-89029-6

**Published:** 2025-02-19

**Authors:** N Mithun, Shamee Shastry, Ganesh Mohan, Jijo Lukose, Murukeshan Vadakke Matham, Santhosh Chidangil

**Affiliations:** 1 Centre of Excellence for Biophotonics, Department of Atomic and Molecular Physics, Manipal Academy of Higher Education, Manipal, 576104 Karnataka India; 2 Department of Immunohematology and Blood Transfusion, Kasturba Medical College Manipal, Manipal Academy of Higher Education, Manipal, 576104 Karnataka India; 3 Department of Atomic and Molecular Physics, Manipal Academy of Higher Education, Manipal, 576104 Karnataka India; 4 Centre for Optical and Laser Engineering, School of Mechanical Aerospace Engineering, Nanyang Technological University, 50 Nanyang Avenue, 639798 Singapore, Singapore

**Keywords:** Raman spectroscopy, Optical tweezers, Platelets, Phospholipids, Platelet activation, Biophysics, Optics and photonics

## Abstract

It is well documented that platelet disorders can result from various causes and can lead to different disease conditions such as cardiovascular diseases (CVDs), thrombocythemia, thrombocytopenia, Autoimmune diseases, Alzheimer’s disease (AD), and even cancer, to name a few. The diagnosis of many of these diseases mainly depends on imaging examinations, clinical analysis and neuropsychological tests, these may be time taking procedures and, have a high chances of false positive/false negative results. The Raman tweezers spectroscopy can provide trust worthy results without much time delay. In the present study the activation dynamics of platelets were studied and observed that the activation leads to biochemical and morphological changes, such as the formation of filopodia on the platelet surface, transformation in the shape from discoid to spherical, and translocation of aminophospholipids from inner leaflet to the outer leaflet of the plasma membrane. The Raman bands corresponding to phospholipids shows remarkable intensity variations during activation. The detailed knowledge regarding the activation dynamics of platelets will be important in monitoring CVDs, ADs, etc. and this paper illustrates a prospective method that can be incorporated into clinical settings in the near future to study and analyze platelet activation. This study will be the first to display the trapping of platelets in its live form to study their activation dynamics using an in-house assembled optical tweezers micro-Raman spectrometer.

## Introduction

Platelet is an indispensable component of blood, and are responsible for blood coagulation. Under normal conditions, platelets will be in an inactive discoid shape^1^. But, when stimulated, these discoid platelets transform into a spherical form, with small projections known as filopodia on their surface^2^. This change indicates the activation of platelets serving as the initial morphological sign of their activation.

Platelet activation usually occur in response to injuries in blood vessels^3^. By receiving stimuli, platelets move toward the wounded area, employing their filopodia to connect with more number of platelets and create a small cluster within the injured blood vessel. This procedure will seal the wound and prevent further bleeding. The process of platelet activation involves various biochemical changes within the platelets. In addition, the activation process consists of the discharge of granules or microparticles from the platelets^4,5^. These microparticles, which are released by the activated platelets, stimulate neutrophils and monocytes through P-Selectin, thereby governing thrombosis and cell interactions. Certain situations may lead to harmful health effects during unwanted platelet activation, with cardiovascular diseases being an important example^6,7^. The peripheral vascular disease, cancer, stroke, and various inflammatory diseases also have relation with increased platelet activation^8,9^. The glycoproteins (CD62, CD63 and PAC-1) expressed on the platelets are considered as the key marker for the activated platelets. The activity of these proteins can cause pathogenesis and coronary heart diseases^10^. When platelets undergo abnormal activation, the substances they release can contribute to the narrowing of arteries in individuals, leading to plaque rupture, thrombus formation, and subsequent ischemic events like myocardial infarction^11^. This occurrence poses a serious threat to the life due to the progression of arterial congestion^12^.

Depending on the physiological circumstances the platelet activation can be considered as both lifesaving as well as life threatening procedure. Exploring the differences between active and inactive platelets is an engrossing research area. The occurrence of these two types in freshly collected platelet-rich plasma lead to a challenge in their isolation and analysis. This can be tackled successfully by optical tweezers micro-Raman spectroscopy technique. This method provides the study of individual live cells. Using an optical microscope and laser beam, it is possible to selectively isolate active and inactive platelets to record their Raman spectra. This Raman analysis offers better knowledge regarding the activation status of platelets. Normally, activated platelets exhibit filopodia formation on their surface, along with a change in shape. The alteration in shape alone cannot be regarded as indicative of active or inactive platelets. There are instances when active platelets can shift back to an inactive state, resulting in a shift from a spherical shape to a discoid form. So, the biochemical changes during this transition also needed to study for the better understanding of these two forms of platelets^8^. This highlights the importance of Raman spectroscopy in distinguishing between active and inactive platelets.

The optical tweezers technique have utilized a tightly focused laser light to hold and manipulate micron and nano-sized dielectric particles^13^. Various groups of researchers have investigated the application of optical tweezers for studying biological samples, including bacteria, viruses, and various live cells^14^. This method offers the advantage of circumventing the need for various chemical fixation methods in single-cell studies. Combining Raman spectroscopy with optical tweezers represents an exceptional method for studying single live cells spectroscopically^15–17^. Optical tweezers are employed to immobilize the moving cells precisely at the laser focus, facilitating the recording of Raman spectra of the cell^18^. Significant research is underway in the domain of optical tweezers and their combination with micro-Raman spectroscopy for studying biological samples^19,20^. Ghanashyam et al. utilized an optical trapping technique involving a vortex beam to conduct an in-depth investigation of the cell membrane^21^. In this method, a spiral phase plate was employed to generate the vortex beam, where the central portion of the laser is removed, resulting in a donut-shaped beam profile. This configuration allows trapping of the cell and specifically probing the membrane, making it possible to obtain detailed information about the RBC membrane. Kato et al. studied single RBCs using mid-infrared photothermal (MIP) microscopy with optical trapping^19^. This vibrational single-cell spectroscopy analysis allowed for the examination of chemical heterogeneities in RBCs, driven by variations in their intracellular properties. Bernecker et al. conducted studies on cell membrane elasticity and deformation using optical tweezers. Their research highlighted the dependence of membrane properties on lipid content. They investigated two different culture conditions for red blood cells (RBCs): one with plasma and another supplemented with human platelet lysate^22^. RBCs cultured with human platelet lysate supplementation exhibited flexibility and morphology closely resembling those of native RBCs. In contrast, RBCs cultured with plasma supplementation displayed a rounded morphology and reduced flexibility. Dorta et al. employed optical tweezers to investigate the membrane rigidity of malaria-infected red blood cells (RBCs) compared to normal RBCs^23^. Their study demonstrated a marked increase in membrane rigidity in the infected cells. Additionally, they analyzed the membrane deformability of infected RBCs in vitro under the influence of commonly used antimalarial drugs, examining both drug-resistant and non-drug-resistant strains of plasmodium falciparum.

When a material is exposed to monochromatic light, it undergoes scattering in two distinct manners: elastic and inelastic. Elastic scattering is the phenomenon where the scattered light retains the frequency of the incident light, whereas inelastic scattering, also known as Raman scattering, involves the scattering of light with varying frequencies^24^. Inelastic scattered light carries energy associated with the molecular vibrations of the sample, thus containing molecular information. Thus, the optical tweezers micro–Raman spectroscopy technique can extract the information on active and inactive platelets.

This study marks the first of its kind to analyze the activation status of platelets using optical tweezers Raman spectroscopy. Flow cytometry studies on individual live platelets are constrained by several limitations. The examination process can potentially harm the platelets, and isolating specific platelets for analysis is challenging due to the rapid flow rate of flow cytometry and the small size of platelets. Sample preparation for flow cytometry is intricate, and the antibodies needed for these studies come at a high cost. Apart from antibodies, specialized equipment and various other reagents are essential for conducting flow cytometry experiments^25^. This highlights the significance of the optical tweezers Raman spectroscopy technique, which provides in-depth information about individual live platelets. As per our knowledge very limited works are reported on single live platelet investigation using micro- Raman combined with optical tweezers^26^.

## Methodology

### Ethics statement

Platelet-rich plasma (PRP) samples were collected from the “Department of Immunohematology and Blood Transfusion, Kasturba Medical College, Manipal, Karnataka, India”, with the approval from the institutional ethics committee (IEC: 68/2018) Kasturba Medical College, Manipal. The informed consent was taken from all the human participants involved in this work. All methods were conducted in accordance with the applicable institutional guidelines and regulations.

## Sample preparation

To achieve the desired suspension of the sample, it is of utmost importance to utilize a suitable physiological medium, considering the substantial quantity of platelets present in PRP. To fulfil this requirement, a platelet additive solution (PAS) was used as the suspension medium. To conduct Raman measurements, 5 µl of PRP was mixed with 1.5 ml of the platelet additive solution. The diluted suspension played a crucial role in facilitating the separation of clustered platelets into individual cells, thereby avoiding the entrapment of multiple platelets in the laser. The experiment employed, 10 mW laser power with 60 s exposure time and accumulation number 2. Raman spectra were recorded from 70 platelets (35 active and 35 inactive platelets) using a lab-assembled optical tweezers Raman spectroscopy system. The laser power density estimated while trapping the single platelet for the current experiment is around 35 mW/µm^2^. The Raman spectra were plotted using Lab spec.6 software integrated with the spectrometer. The smoothing and interpolation were performed using Origin software, while baseline correction and vector normalization were carried out in MATLAB. Using processed data, the intensity variation of certain Raman bands, especially phospholipid, phenylalanine, tyrosine, and tryptophan, were investigated in the respective spectra of active and inactive platelets, which shows remarkable intensity variations. The Principal Component Analysis (PCA) was used to classify the Raman spectra of active and inactive platelets. Principal Component Analysis (PCA) is a dimensionality reduction technique that transforms data into a new set of variables called principal components. These components are designed to capture the maximum variance in the data while reducing its dimensional complexity. By identifying patterns in high-dimensional datasets, PCA projects the data onto a lower-dimensional space, retaining the most significant information.

## Apparatus

**Fig. 1:**
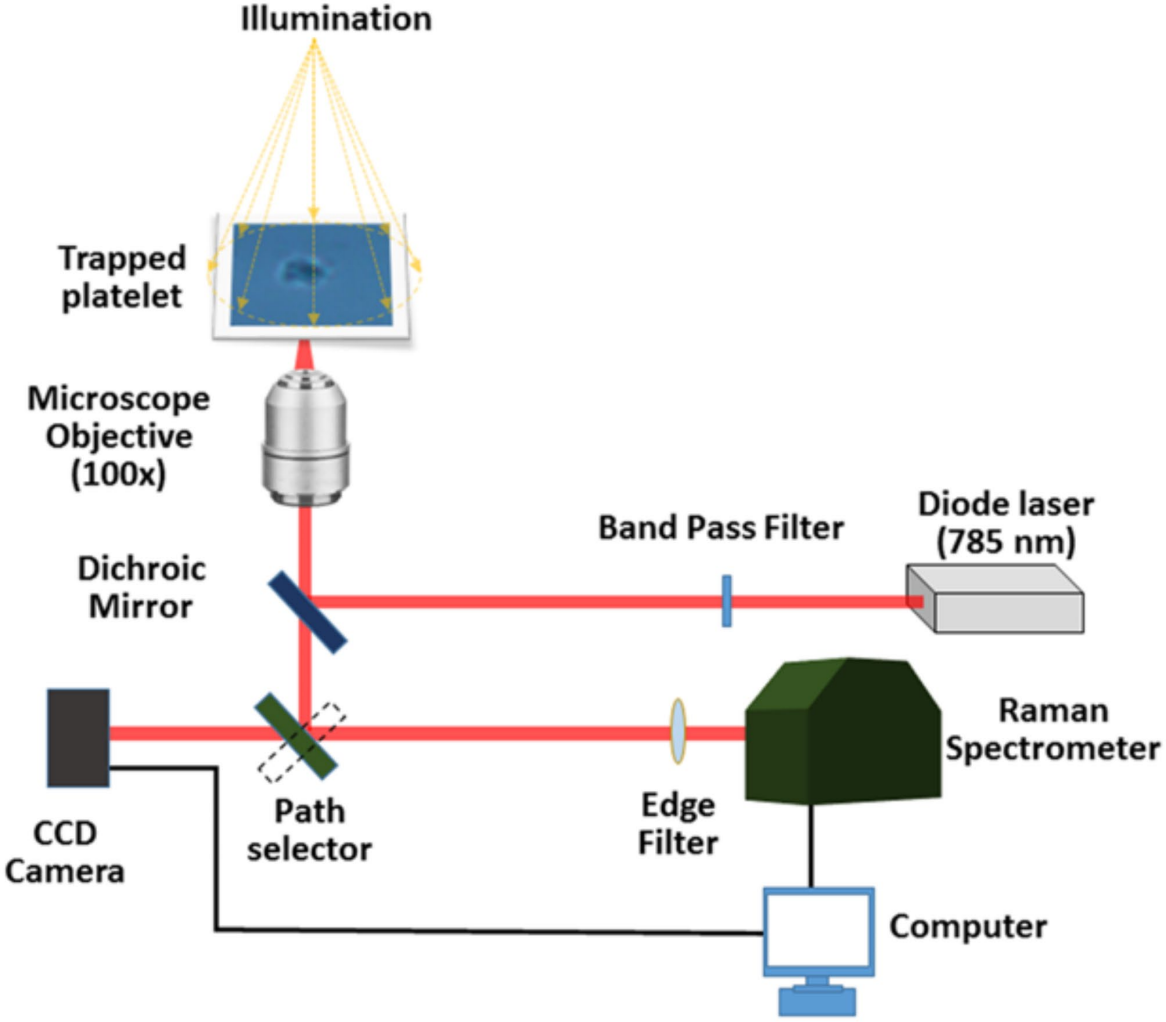
Schematic of the lab-built Optical Tweezers Raman spectroscopy setup.

The lab-assembled optical tweezers micro-Raman spectroscopy system is depicted in Fig. 1. The system utilizes a 785 nm diode laser for both tweezing and recording the spectra. The laser has an additional wavelength near the 790 nm range. A bandpass filter is used to eliminate this wavelength and provide only the 785 nm wavelength. The dichroic mirror in the setup reflects the 785 nm laser beam and transmits Raman signal above 785 nm. In the optical tweezers setup, an inverted microscope enables the laser beam to focus from the bottom of the sample holder to create the laser trap. The 100x microscope objective is utilized to direct light onto the sample and collect backscattered Raman signal. The scattered signal passes through the dichroic mirror and reaches an optical path selector in the microscope. This optical path selector directs the signal to the spectrometer for recording Raman spectra and to the CCD camera for capturing microscopic images of the sample. The Raman scattered light was filtered from Rayleigh scattered signals using an edge filter. The spectrometer used for the study has the gratings with 1200 groves per millimeter blazed at 750 nm.

## Results and discussion

### Raman band assignment of single live platelet

**Fig. 2:**
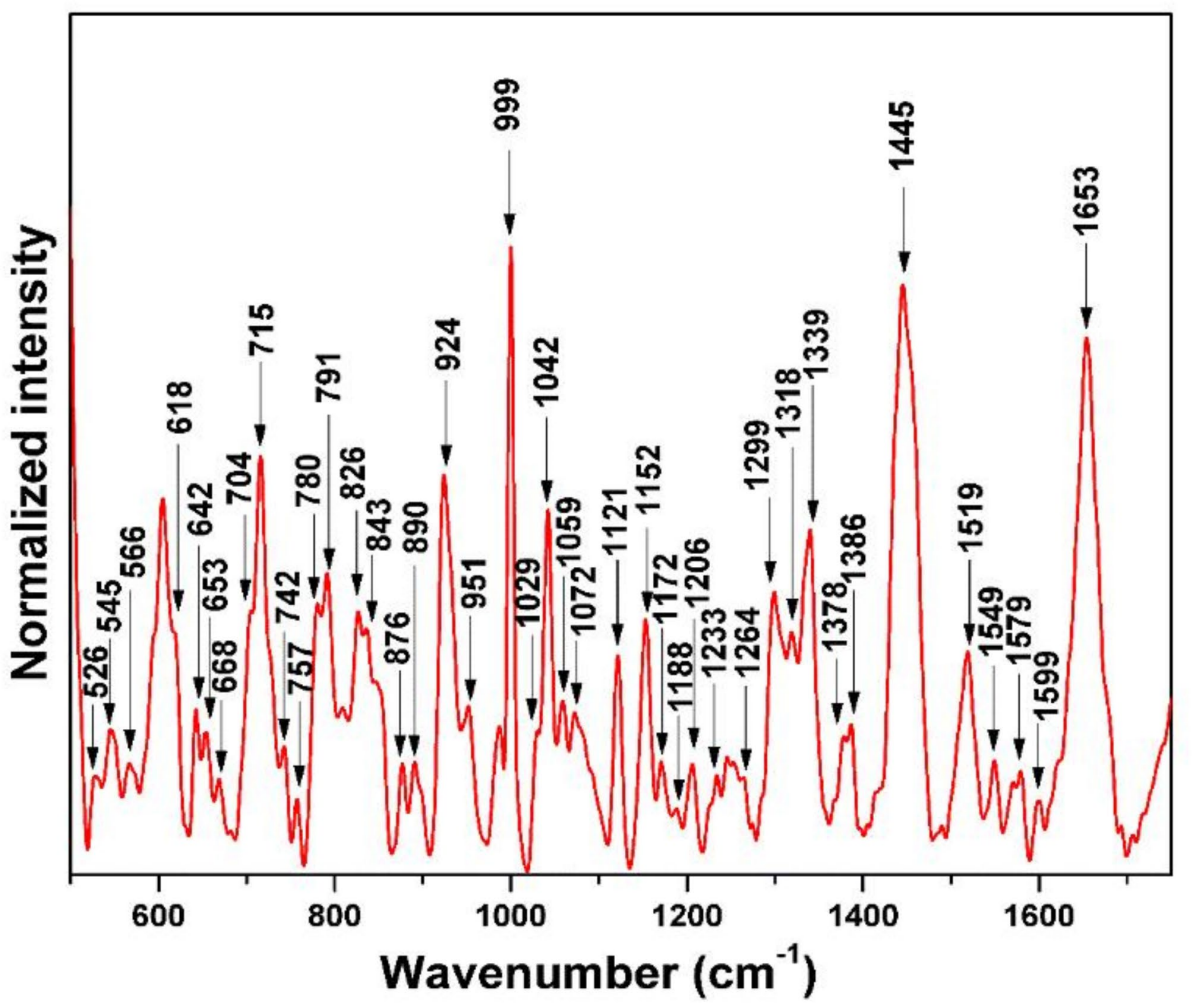
Raman spectrum and band assignment of optically trapped single live platelet suspended in platelet additive solution (PAS).

The vector normalized Raman spectrum of single live platelet suspended in Platelet additive solution (PAS) is given in Fig. 2, and the band assignments are given in Table 1. The Raman bands of platelets primarily originate from various proteins, amino acids, and lipids^27^. The Raman spectra of individual viable platelets have an intense band at 999 cm^− 1^, attributed to the phenylalanine symmetric ring breathing (amino acid). Similarly, the peak at 1445 cm^− 1^ (lipid) resulting from the CH_2_ bending exhibits a higher intensity, while the peak at 1653 cm^− 1^ (protein) due to the C = C stretch of amide I also displays a high intensity compared to many Raman peaks observed in the spectrum. Other prominent bands include 704 cm^− 1^ from the C-S stretching, 715 cm^− 1^ from the choline C-N symmetric stretch, 791 cm^− 1^ from the amino acid, 924 cm^− 1^ from C-C stretch, 1042 cm^− 1^ from the C-N stretching of polypeptide, 1121 cm^− 1^ from the C-C stretch of phospholipid, 1152 cm^− 1^ due to the C-C stretch of beta carotene, 1299 cm^− 1^ due to the CH_2_ twist, 1339 cm^− 1^ is from the C-H bend of phenylalanine and tryptophan, and 1519 cm^− 1^ due to the C-C stretch of beta carotene. Apart from 1121 cm^− 1^ and 1445 cm^− 1^, less intense peaks of phospholipids such as 1059 cm^− 1^ (trans-configuration C-C stretching), 1436 cm^− 1^ corresponds to CH_2_ bending were also present^28–30^.

**Table 1:** The Raman band assignment of single live platelet^27–35^.

Raman bands (cm^− 1^)	Vibration	Component
526	δ(C–CO), δ_ring_ (in plane bending)	Phenylalanine, Tyrosine, Tryptophan
545	S-S stretch vibrations	Cysteine
566	-------------	Tryptophan
583	-------------	Glucose
618	C-C twist	Phenylalanine
642	C-C twist	Tyrosine
653	-------------	Tyrosine
668	C-S stretch	Cysteine
680	C-S Vibrations	……………
704	C-S stretch	……………
715	C-C-N^+^ symmetric in	Phosphatidylcholine
742	O-P-O symmetric stretch	Tryptophan
757	Phosphate diester stretch	Phosphatidylethanolamine
780	O-P-O diester symmetric stretch	…………….
791	-----------------	Amino acids
808	-----------------	Tryptophan
826	O-P-O diester anti-symmetric stretch	Tyrosine
843	-----------------	Tyrosine
865	C-C backbone vibration	Proteins, Lipids
876	v(C-C)	…………….
924	C-C stretch	…………….
951	C-C backbone vibration	Proteins
987	--------------	Amino acids
999	C-C sym. ring breathing	Phenylalanine
1029	Inplane C-H bending	Phenylalanine
1042	C-N stretch	Polypeptide
1059	C-C trans stretch	Phospholipid
1072	C-C gauche stretch	Lipid
1082	C-N stretch	Phospholipid
1097	C-C gauche stretch	Phospholipid
1114	v(C-N)	…………….
1121	C-C trans stretch	Phospholipid
1152	C-C stretch	β-Carotene
1172	In-plane C-H bending with ring stretching,	Phenylalanine/Tyrosine
1188	C-C skeletal stretch	………………
1206	C-C stretch	Tyrosine, Phenylalanine
1233	PO_2_ Anti-symmetric stretch	………………
1245	-----------------	Protein (amide III)
1258	-----------------	Tryptophan
1264	------------------	Phospholipid, Protein (amide III)
1284	------------------	Protein (amide III)
1299	CH_2_ twist	Phospholipid, Protein (amide III),
1318	CH_2_ twist	…………….
1339	Aromatic ring mode, C-H bend	Tryptophan, Phenylalanine
1378	CH_2_ wagging	………………
1386	CH_2_ wagging	………………
1404	COO^−^ symmetric stretch	………………
1419	CH_3_ asymmetric stretch	Lipids
1436	CH_2_ bend	Phospholipid
1445	CH_2_ bend	Phospholipid
1455	CH_2_ bend	Lipids
1506	C = C stretch	…………….
1519	C = C stretch	β-Carotene
1549	---------------	Polypeptide
1570	---------------	Tryptophan, Phenylalanine
1579	Aromatic ring mode	Tryptophan
1599	Bond stretch (v) C = O,	Phenylalanine
1612	Aromatic ring mode	Tyrosine
1653	C = C stretch	Protein (amide I), Cholesterol,

Note: --------------: vibrational assignment unknown ……………: Component unknown.

### Microscopic images of inactive and active platelets

**Fig. 3:**
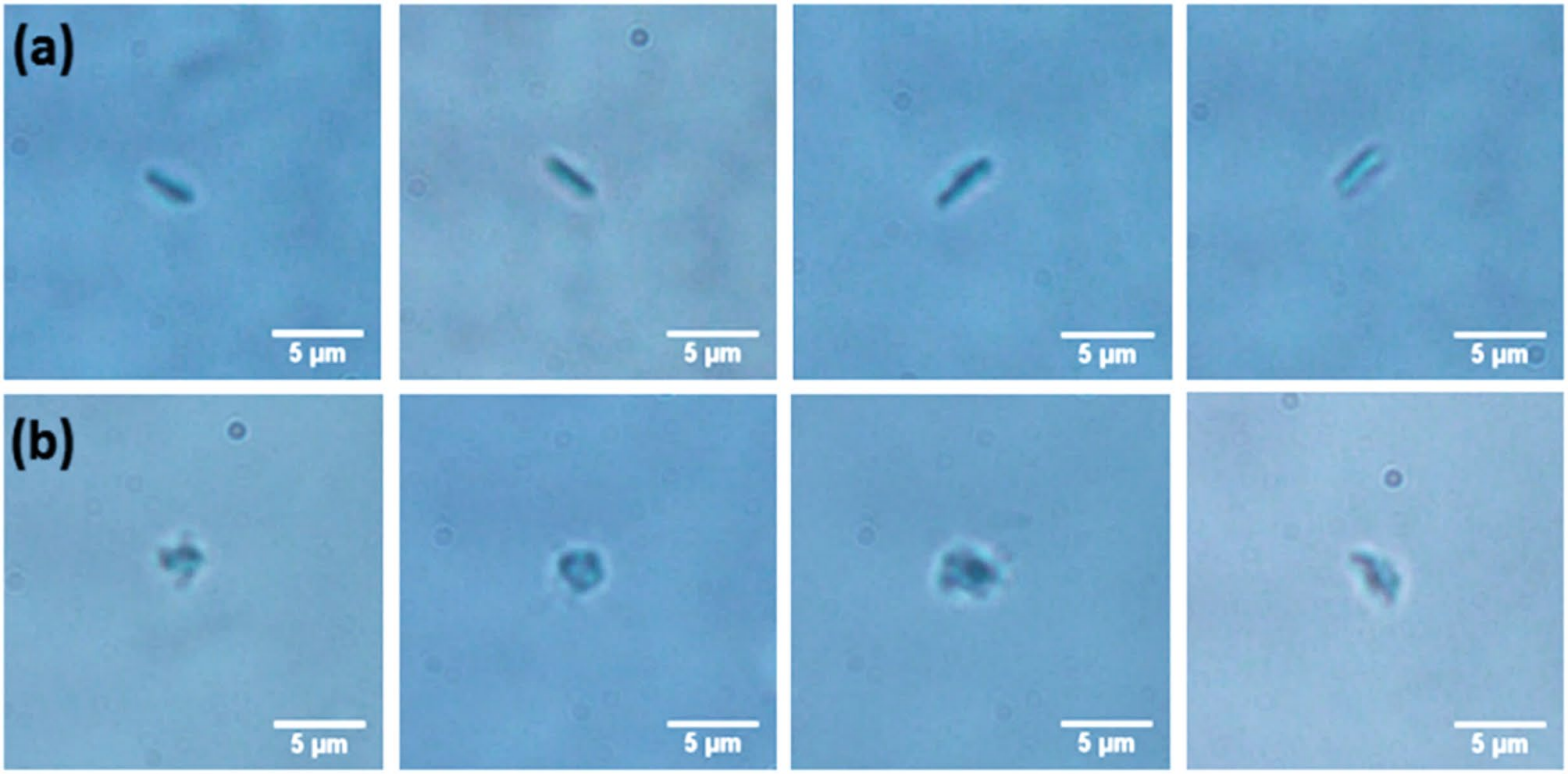
Microscopic images of (**a**) inactive platelets and (**b**) active platelets.

The microscopic images of inactivated and activated platelets are shown in Fig. 3. The inactive platelets display a discoid shape. The side view of these inactive platelets are shown in Fig. 3a, revealing the absence of filopodia formation and a non-spherical shape. The activated platelets exhibit a spherical shape and filopodia formation, as depicted in the microscopic images (Fig. 3b). The filopodia helps to interconnect each platelets, and also help to stabilizing the blood clot.

### Comparison of the Raman spectra of inactive and active platelets

**Fig. 4:**
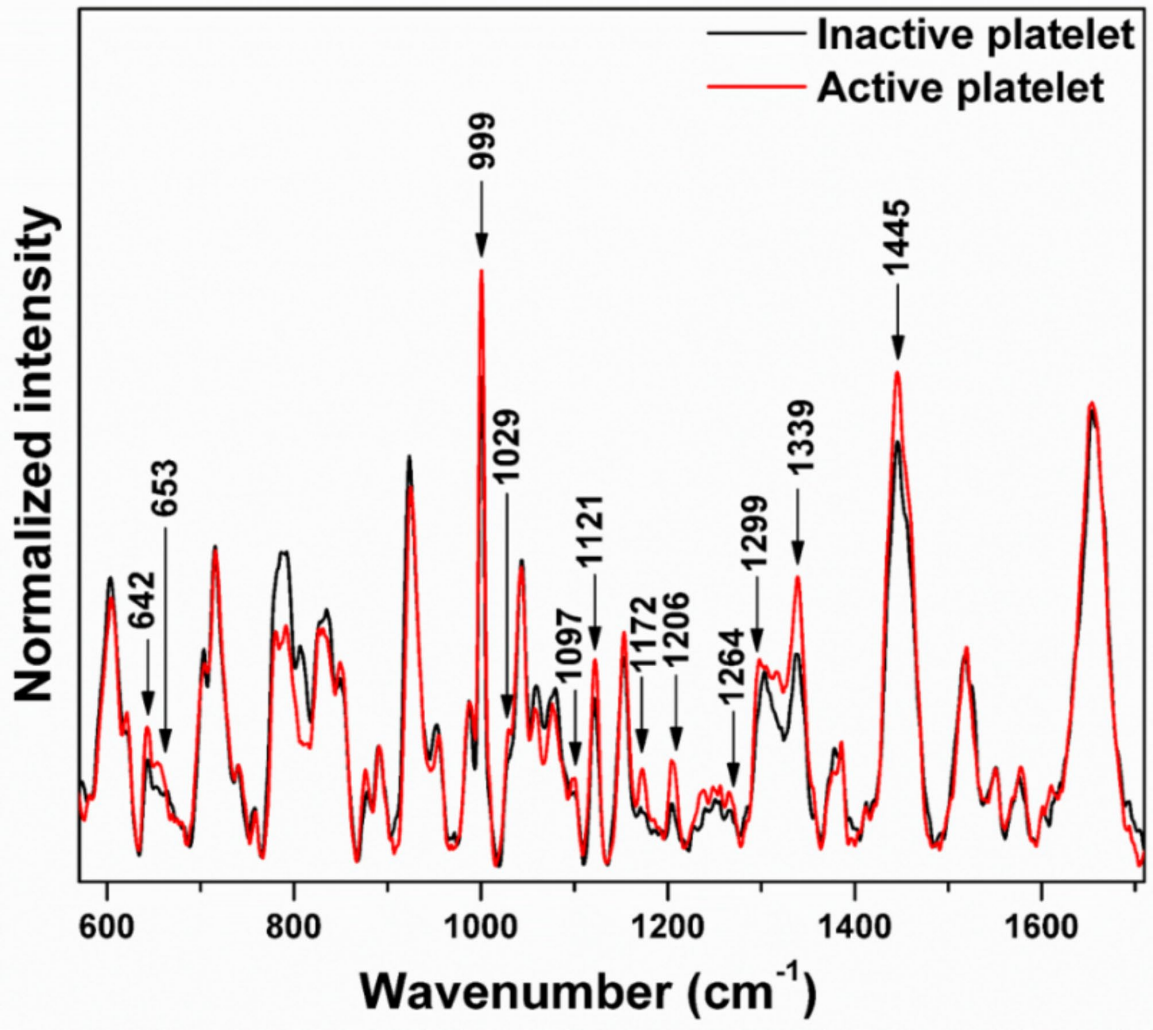
Raman spectra of inactive and active platelets.

Figure 4 illustrates the significant variations in the Raman band intensity of inactive platelets in comparison with the active platelets.

Both spectra displayed in the Fig. 4 represent the average of 35 individual spectra, each recorded from different platelets. The significant variations were contributed by the characteristic peaks of phospholipids and amino acids.

### Phospholipid bands

**Fig. 5:**
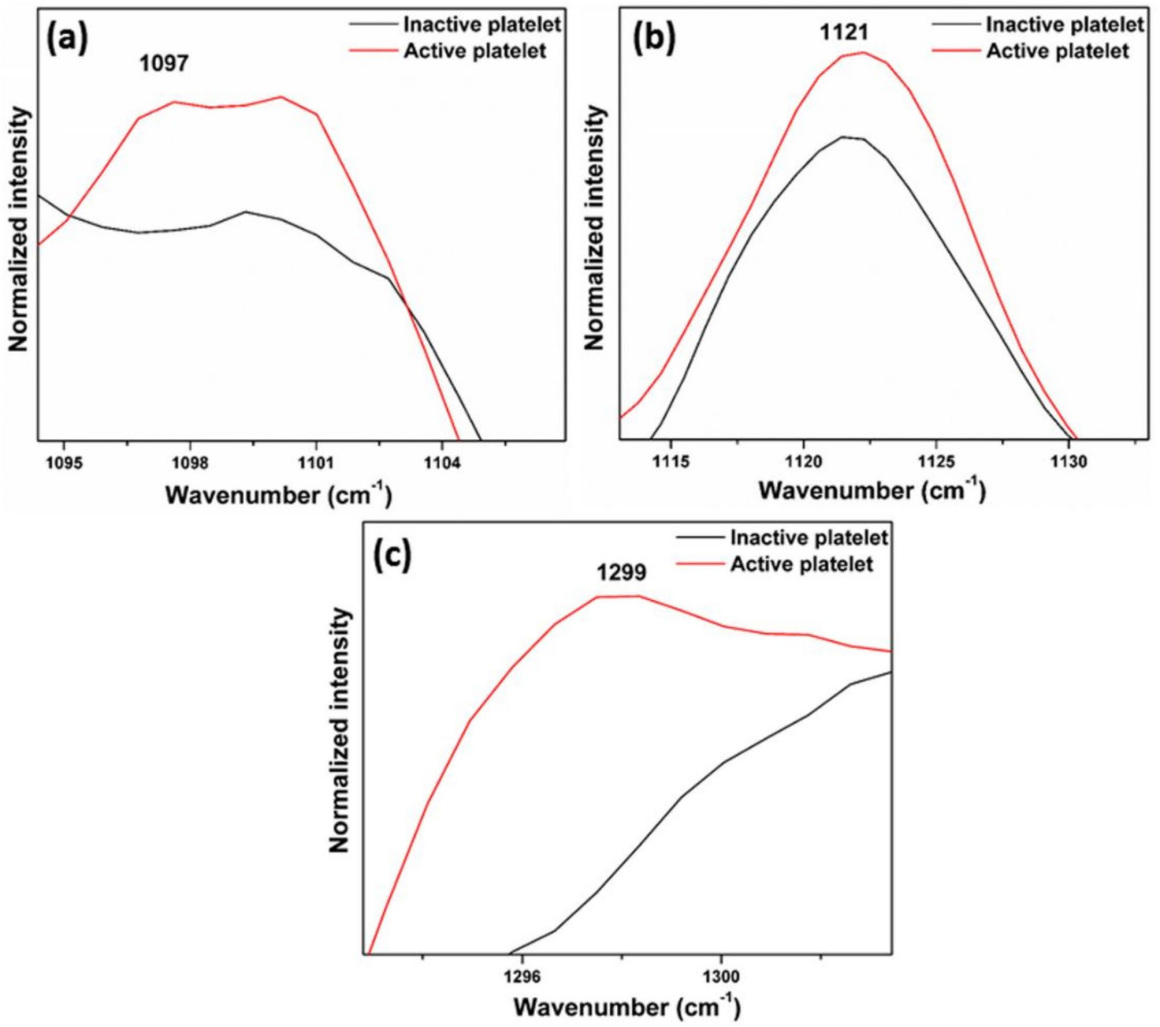
Expanded regions (Phospholipid bands) of the Raman spectra (Fig. 4) of inactive and active platelets at (**a**) 1097 cm^− 1^, (**b**) 1121 cm^− 1^, and (**c**) 1299 cm^− 1^.

**Fig. 6:**
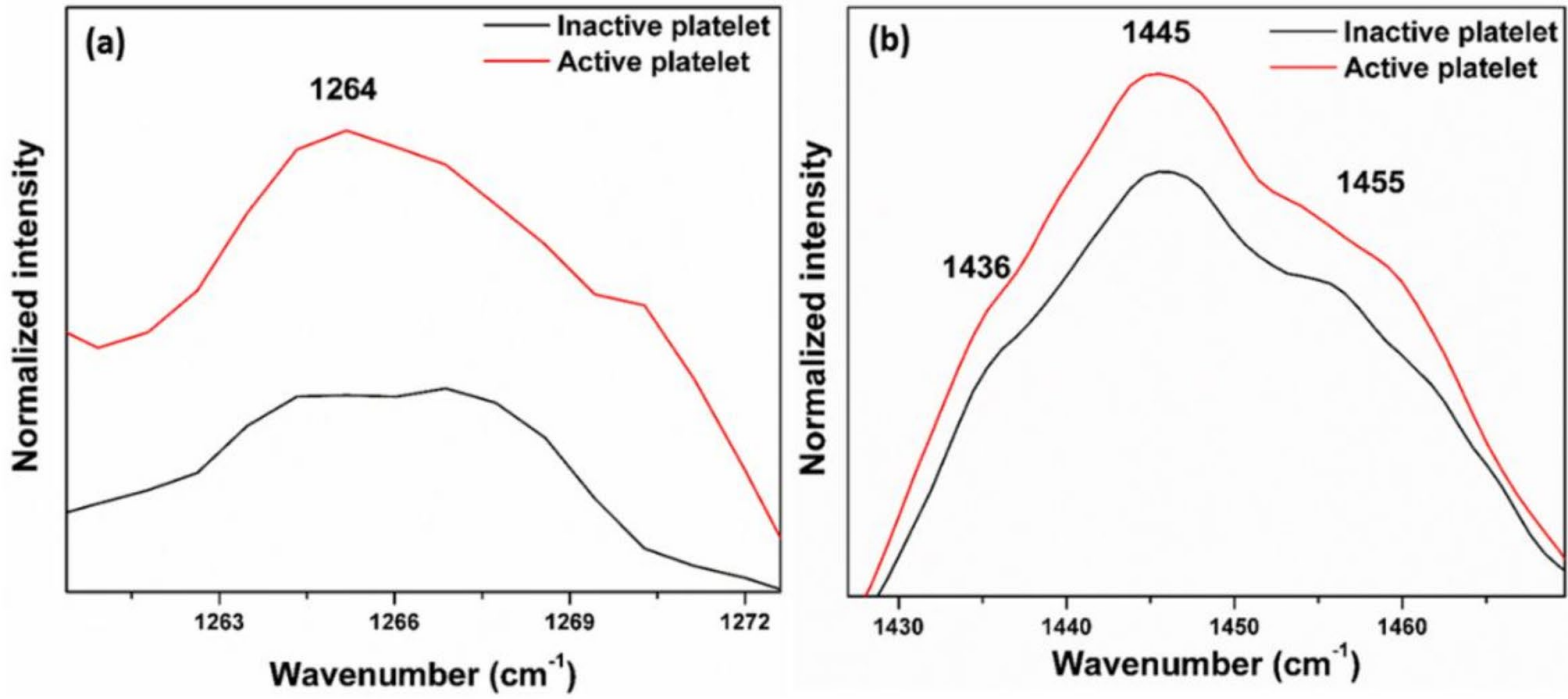
Expanded regions (Phospholipid bands) of the Raman spectra (Fig. 4) of inactive and active platelets (**a**) 1264 cm^− 1^ (**b**) 1436 cm^− 1^, 1445 cm^− 1^, and 1455 cm^− 1^.

The Raman spectra shown in Figs. 5a-c and 6 are expanded regions from Fig. 4 at the respective wavenumbers to reveal increased intensities in active platelets for various lipid bands especially phospholipid bands including 1097 cm^− 1^ (C-C gauche stretch), 1121 cm^− 1^ (C-C trans. stretch), 1299 cm^− 1^ (CH_2_ twisting), and 1264 cm^− 1^  , alongside others such as 1436 cm^− 1^, 1445 cm^− 1^, and 1455 cm^− 1^ (CH_2_ bend). These findings strongly suggest that the membranes of active platelets contain a higher concentration of phospholipids. This increased expression of phospholipids is reflected in the increment in intensity of the Raman bands associated with phospholipids. The proteins (glycoproteins) and phospholipids will be expressed by the activated platelets on their membrane surface^36^. The exterior layer of the platelet plasma membrane is predominantly made up of choline phospholipids, specifically sphingomyelin and phosphatidylcholine^36^. In contrast, the inner layer primarily consists of aminophospholipids, specifically phosphatidylserine and phosphatidylethanolamine^37,38^. When activated, translocation occurs, causing the aminophospholipids to move from the inner layer to the outer layer of the membrane^39–41^. Thus, the phospholipid concentration in the outer leaflet of the plasma membrane in active platelets has elevated^42,43^. The cytoskeleton of platelets is primarily made up of actin, tubulin, spectrin, and filamin^44^. In the case of inactive platelets, microtubule coils are composed of αβ-tubulin polymers, which are situated below the plasma membrane, preserving the discoid shape^45,46^. When exposed to different biological signals and agonists, alterations in shape occur as a result of the disassembly and rearrangement of the cytoskeletal components^46^. When the shape change occurs, the surface of the platelets forms small projections called filopodia, which are formed from the core actin fibers^47^.

### Phenylalanine and tryptophan bands

**Fig. 7:**
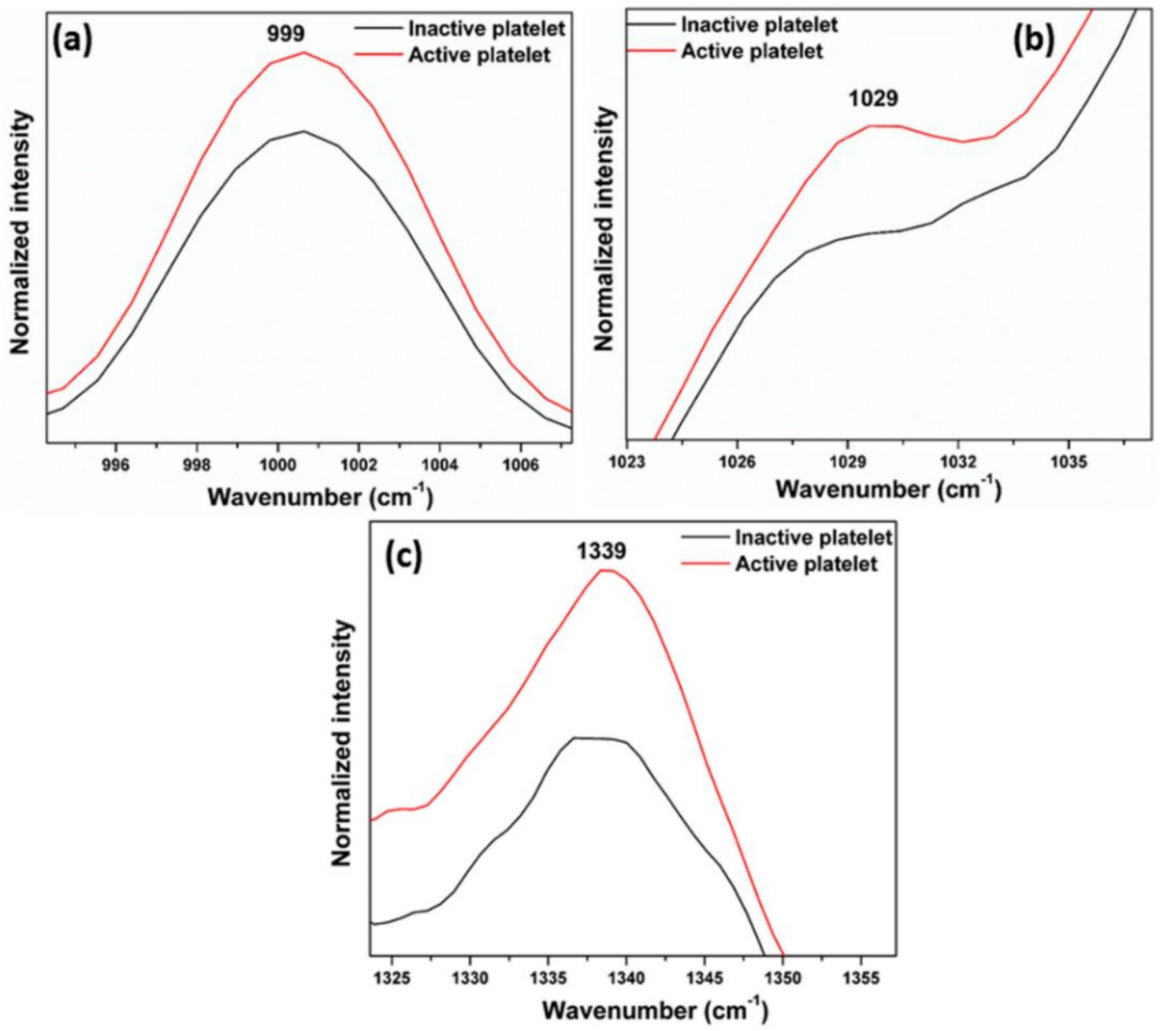
Expanded regions: (**a**,** b**) Phenylalanine band, and (**c**) Tryptophan (1339 cm^− 1^) bands (Fig. 4) of inactive platelets, and active platelets.

As evident from Fig. 7, the intensity of the 999 cm^− 1^ peak by the symmetrical ring breathing of phenylalanine, and 1029 cm^− 1^, due to the inplane C-H bending of phenylalanine, are higher in active platelets. Since phenylalanine is present in glycoproteins, the activation of platelets leads to the expression of these proteins on the surface of active platelets, which may result in a change in phenylalanine concentration on the membrane. Notably, the tryptophan peak at 1339 cm^− 1^ (aromatic ring mode) displays a discernible difference in intensity between active and inactive platelets. Moreover, the increased intensity of tryptophan might be due to the expressed proteins on the active platelet surface.

### Tyrosine bands

**Fig. 8:**
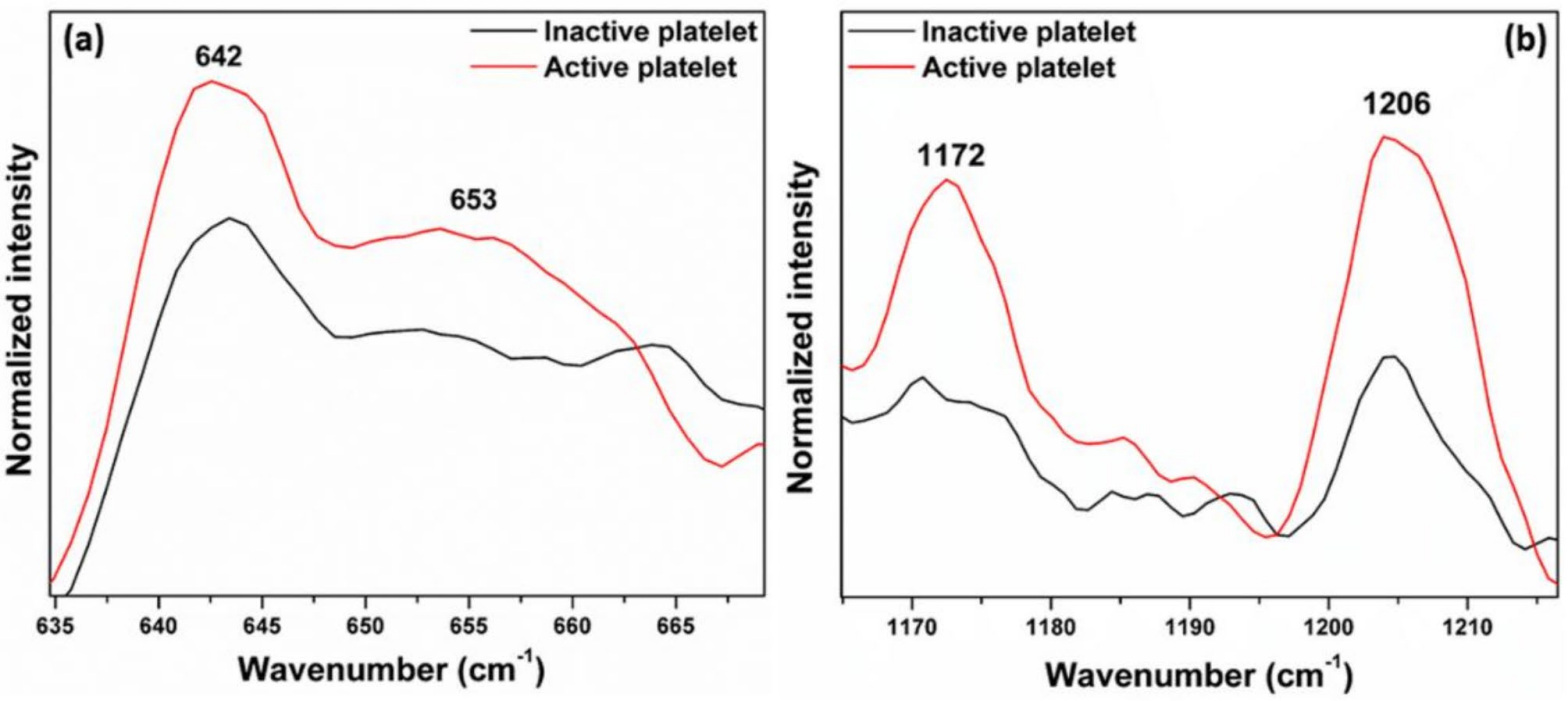
Expanded regions: (**a**,** b**) Tyrosine bands (Fig. 4) of inactive and active platelets.

Figure 8 displays the variation in the intensity of tyrosine bands. The Raman bands of tyrosine, specifically at 1172 cm^− 1^ (in-plane C-H bend combined with ring stretching), 1206 cm^−^ ^1^ (C-C stretch), 642 cm^− 1^  (C-C twist), and 653 cm^− 1^, exhibit higher intensity in active platelets. Platelet activation triggers multiple signaling pathways, resulting in phosphorylation events and the activation of tyrosine kinases^48,49^. These kinases phosphorylate tyrosine residues on proteins, such as membrane receptors and intracellular signaling molecules have a pivotal role in transmitting activation signals and facilitating platelet aggregation. However, it is worth noting that the changes in tyrosine concentration can vary depending on several factors, including the specific activation stimulus, as well as the duration and intensity of activation.

The polypeptide backbone of P-selectin consists of amino acids, such as tyrosine, that are connected by peptide bonds; in addition to other amino acids, tyrosine has a vital role in the structure and function of P-selectin. The P-selectin on the activated platelet surface could lead to an increase in the intensity of tyrosine bands. So the expression of glycoproteins may be responsible for changes in tyrosine intensity in the Raman spectra of activated platelets^50^. The increased intensity of tyrosine, phenylalanine, and tryptophan observed in Raman spectra of active platelets suggest the expression of various proteins, particularly glycoproteins, on the platelet surface.

### Principal component analysis

**Fig. 9:**
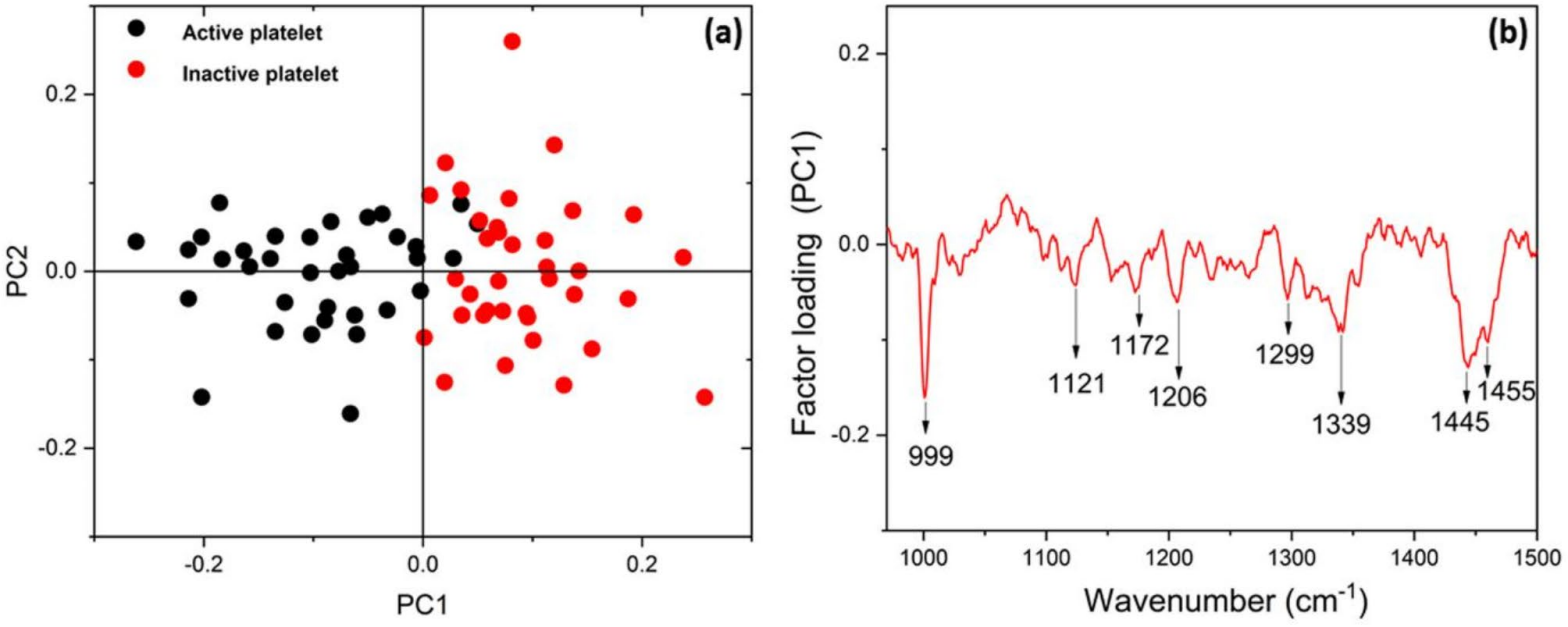
(**a**) PCA scattering plot of active and inactive platelet (**b**) Factor loading plot.

Principal Component Analysis (PCA) was carried out on the Raman spectra of both inactive and active platelets. The PCA score plot shown in Fig. 9a indicates a distinct separation between the two platelet conditions. This plot utilizes principal components PC1 and PC2 and focuses on the spectral range of 970 cm^− 1^ to 1500 cm^− 1^ for the analysis. This particular region is primarily characterized by phospholipid bands present in platelet spectra. Figure 9b presents the factor loading plot, indicating the key Raman bands responsible for discrimination between active and inactive platelets. Prominent Raman bands contributing significantly to the clustering of the data include those at 999 cm^− 1^, 1121 cm^− 1^, 1172 cm^− 1^, 1206 cm^− 1^, 1299 cm^− 1^, 1339 cm^− 1^, 1436 cm^− 1^, 1445 cm^− 1^, and 1455 cm^− 1^.

### Platelet activation induced by the external addition of thrombin

#### Same platelet before and after thrombin addition

The platelets were intentionally activated using thrombin to confirm the Raman spectra of active and inactive platelets. Thrombin activates platelets by binding to protease-activated receptors (PARs) on their surface, primarily PAR-1 and PAR-4 in humans^51^. This activation mechanism involves the proteolytic cleavage of the receptors, unveiling a new amino-terminal sequence that functions as a tethered ligand to initiate intracellular signaling cascades. The signaling triggered by thrombin leads to cytoskeletal remodeling in platelets, resulting in their transformation from the discoid to spherical form with filopodia formation. Additionally, thrombin stimulation promotes the expression of glycoproteins and phospholipids on the platelet surface^52,53^.

**Fig. 10:**
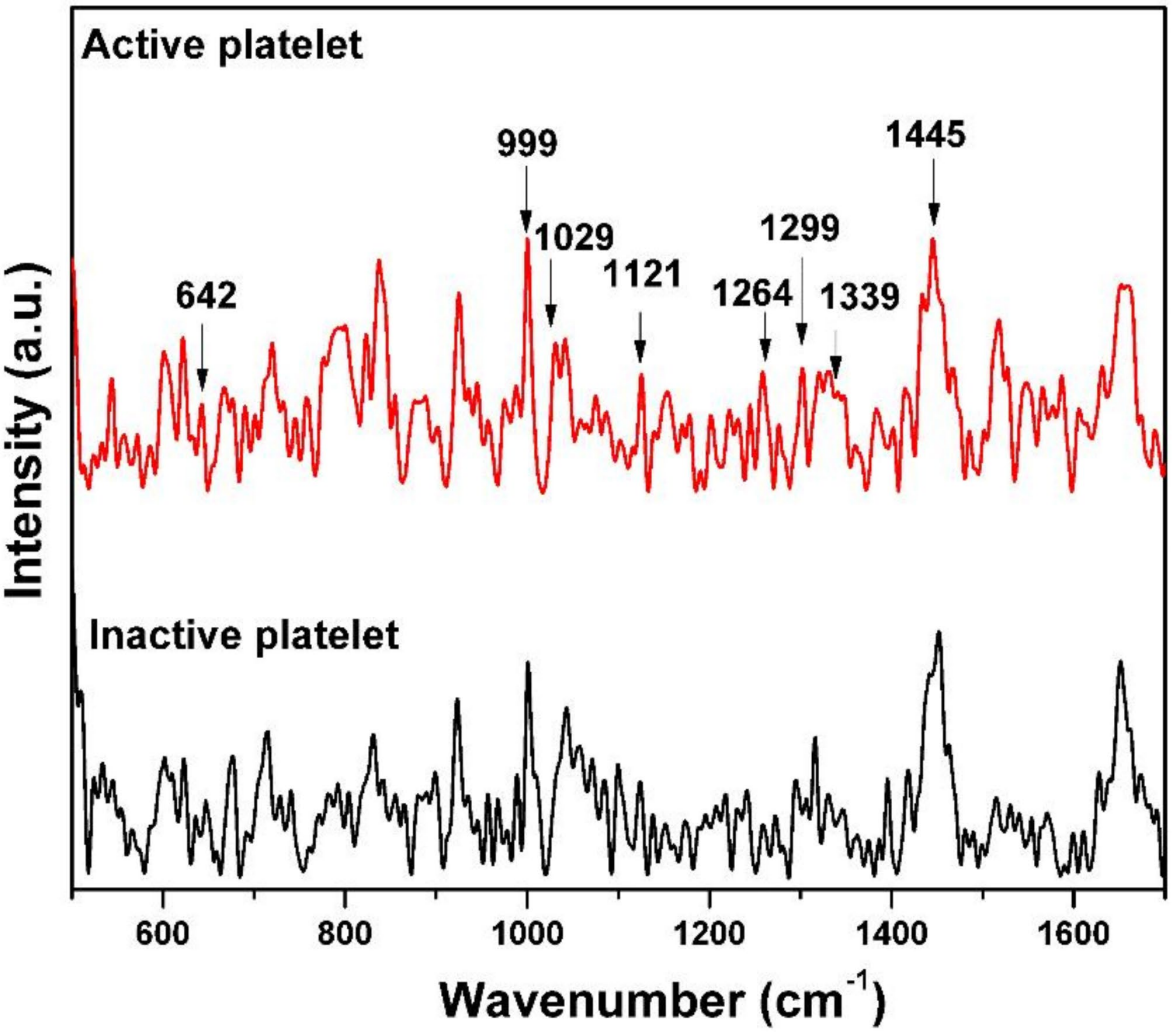
The Raman spectra of the same single platelet before activation (before thrombin addition) and after activation (after thrombin addition).

**Fig. 11:**
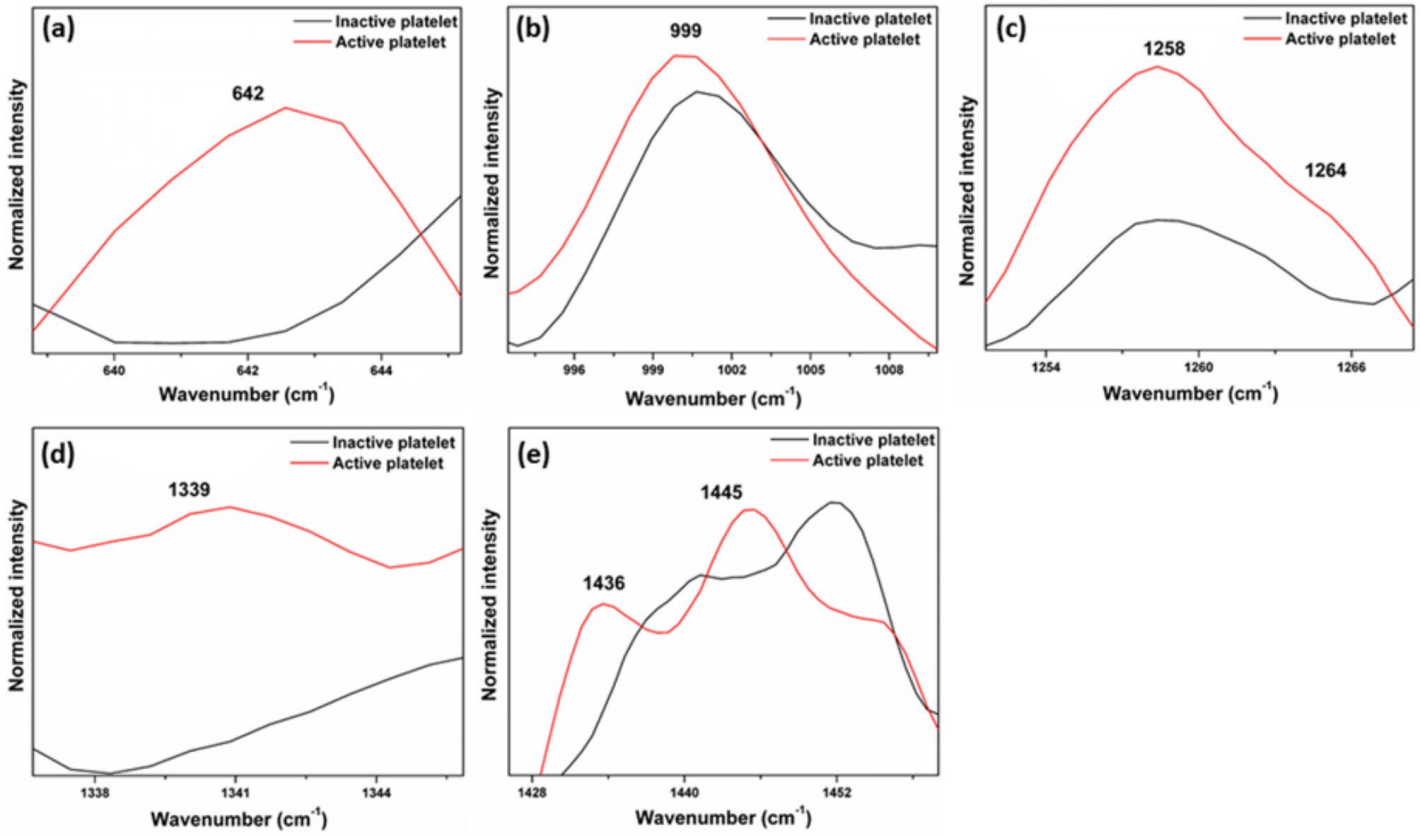
Expanded regions of the Raman spectra of inactive (before thrombin addition) and active platelets (after thrombin addition).

Freshly collected platelets were suspended in a platelet additive solution, and a single inactive platelet was trapped. The Raman spectrum was acquired from the trapped platelet, after recording the spectrum, 10 µl of thrombin was added to the sample well using a pipette to activate the trapped platelet. The same platelet was kept in the laser spot during the thrombin addition to make sure that the Raman spectra were recorded from the same inactive and active platelet. After adding thrombin the platelet was released from the laser trap for avoiding the photodamage caused by prolonged laser beam exposure. After releasing the platelet from the laser trap its movement was monitored under the microscope. Once the platelet changed shape from discoid to spherical with filopodia formation, the same platelet was trapped again. The recorded spectra are presented in Fig. 10, with some expanded regions of the Raman spectra displayed in Fig. 11.

The Raman spectrum of platelets after the addition of thrombin reveals characteristic signs of activation, with increased Raman peak intensities in the regions associated with phospholipids, phenylalanine, tryptophan, and tyrosine. Notably, the Raman band at 642 cm^− 1^ (Fig. 11a), corresponding to the C-C twisting mode of tyrosine, shows higher intensity in activated platelets. Similarly, phenylalanine bands at 999 cm^− 1^ (C-C symmetric ring breathing) (Fig. 11b), and 1029 cm^− 1^  (Inplane C-H bending) display enhanced intensities upon activation. The tryptophan band at 1339 cm^− 1^ (Fig. 11d), linked to the aromatic ring vibration mode, and 1258 cm^− 1^ (Fig. 11c) also exhibits greater intensity in the activated state. Additionally, phospholipid bands at 1121 cm^− 1^, 1264 cm^− 1^, 1299 cm^− 1^, 1436 cm^− 1^, and 1445 cm^− 1^ (Fig. 11e) show significant intensity increase after activation.

**Fig. 12:**
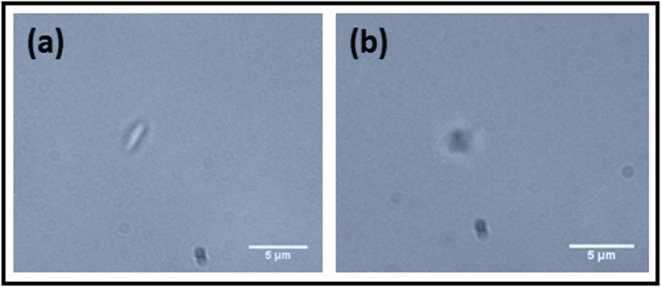
Microscopic images of a platelet (**a**) before thrombin addition, (**b**) same platelet after thrombin addition.

The microscopic images of inactive platelets (before thrombin addition) and after activation (after thrombin addition) are shown in Fig. 12 (a, b). It shows that the platelet before the thrombin addition had a discoid shape, and there was no filopodia formation, but after the thrombin addition, the platelet became active, and the discoid shape was changed to a spherical shape with filopodia on the platelet surface.

The Raman spectra of thrombin-treated activated platelets and naturally activated platelets show a similar trend. The phospholipid bands are showing increased intensity in both cases. The phenylalanine band, tyrosine bands and tryptophan bands are also showing increased intensity in both cases.

#### Randomly selected platelets before and after thrombin treatment

**Fig. 13:**
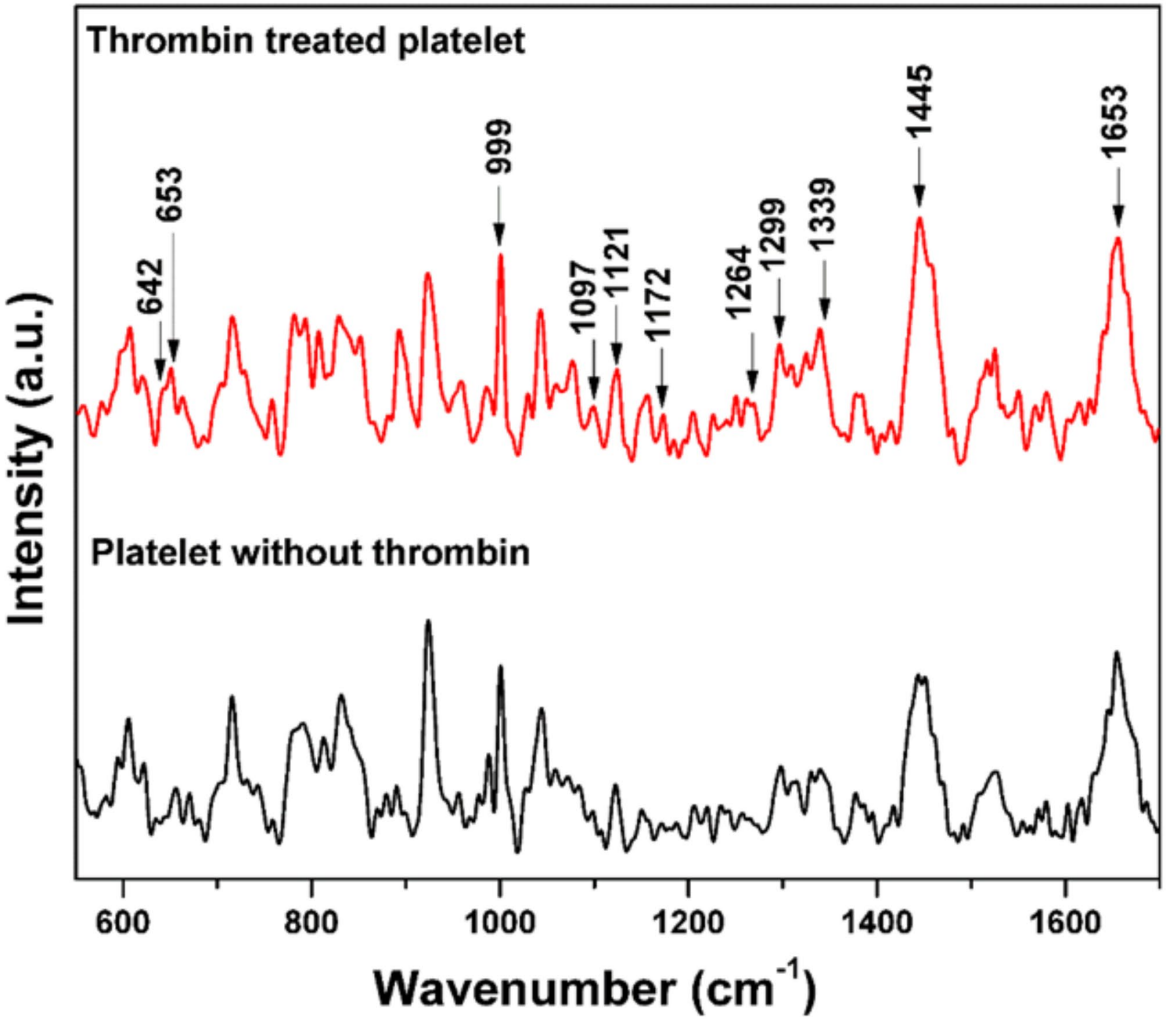
The Raman spectra of platelets before treatment (without thrombin) and after treatment (with thrombin).

To activate platelets, 10 µL of thrombin was added into the PAS solution in which the platelets were suspended. The Raman spectra were recorded from both the control sample (PAS without thrombin) and the thrombin-treated sample. In the thrombin-treated solution, all the platelets were in their activated state. Raman spectra were recorded from thrombin-treated active and untreated inactive platelets. The corresponding average Raman spectra are shown in Fig. 13.

Similar to the Raman spectra observed for naturally activated platelets, the thrombin-treated platelets were also shows increased Raman band intensities associated with phospholipids and proteins. As shown in Fig. 5, the intensities of phospholipid bands at 1097 cm^− 1^, 1121 cm^− 1^, 1264 cm^− 1^, 1299 cm^− 1^, 1436 cm^− 1^, 1445 cm^− 1^, and 1455 cm^− 1^ are notably higher in thrombin-treated platelets, reflecting their activation state. Thrombin is widely recognized for directly activating platelets by engaging protease-activated receptors (PARs), specifically PAR-1 and PAR-4 ^51^. So, this investigation shows that the expression of proteins and phospholipids on the platelet membrane is due to the activation of platelets.

#### Investigation on the laser induced photodamage of platelets

**Fig. 14:**
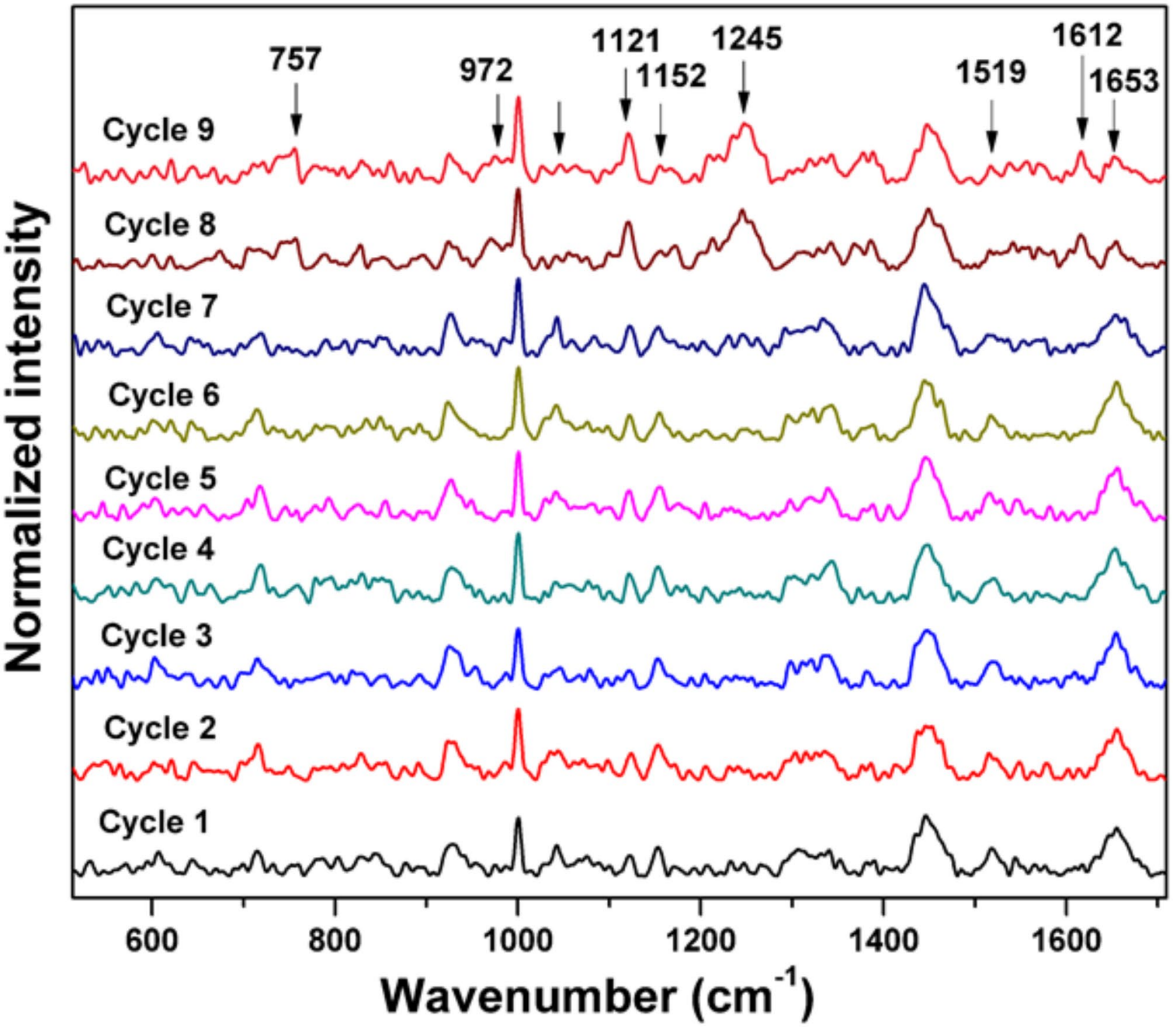
The Raman spectra of a single platelet recorded 9 times consecutively.

**Fig. 15:**
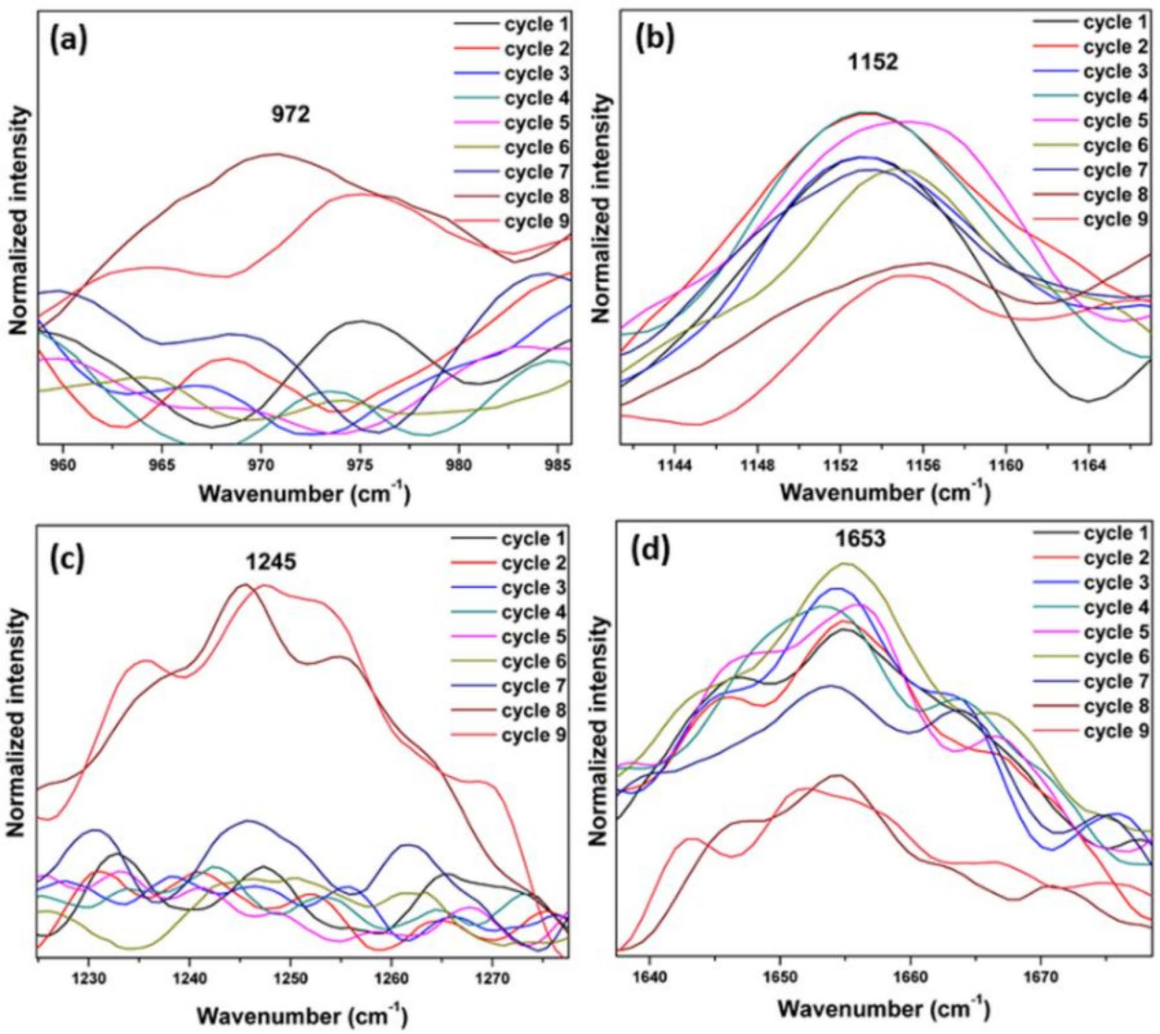
The expanded region of Raman band (**a**) 972 cm^− 1^, (**b**) 1152 cm^− 1^, (**c**) 1245 cm^− 1^, and (**d**) 1653 cm^− 1^.

To investigate photodamage in optically trapped live platelets, the laser trap was maintained for an extended period, and the Raman spectra were repeatedly recorded from the same platelet. The recorded Raman spectra of platelet with a 785 nm laser excitation, 10 mW laser power (power density ~ 35 mW/µm^2^), 60s exposure time and 2 accumulations are given in Fig. 14. If any possibility of photodamage occurs, there will be a remarkable increase in the intensity of Raman bands at 972 cm^−1^, and 1245 cm^−1^. The increased intensity of Raman peaks at 1245 cm^−1^ and 972 cm^−1^ are due to the heat induced protein denaturation (Fig. 15 (a) and Fig. 15 (c)) ^[27^. The expanded region of Raman bands at 972 cm^−1^, 1152 cm^−1^, 1245 cm^−1^, and 1653 cm^−1^ are shown in Fig. 15 (a-d). The beta-carotene band at 1152 cm^−1^ (Fig. 15b) and 1519 cm^−1^ have reduced intensity with more laser irradiation time. There is an increased intensity of 1612 cm^−1^ (tyrosine) band observed during 8th and 9th cycle. The amide I band at 1653 cm^−1^ (Fig. 15 (d)) has intensity reduction due to longer laser irradiation. Similar results were also observed in the photodamage study conducted by Surekha et al. in red blood cells ^27^. In another similar investigation the 972 cm^−1^ band was identified as one of the characteristic Raman marker band for identifying heat induced photodamage of the cells ^54^. In the current experiment, the Raman spectrum recorded from a live platelet took a maximum of 6 min, with no signs of photodamage observed. However, clear evidence of photodamage was found when the 8th and 9th spectra were recorded at 48 and 54 min, respectively, from the same platelet. This indicates that it is safe to record the Raman spectra of platelets with the optimized parameters 10 mW laser power, 60s exposure time and two accumulations for the present study.

## Conclusions

An in-house developed optical tweezers micro-Raman spectrometer is used to trap platelets and illustrate the activation dynamics of platelets. In comparison to inactive platelets, Raman bands observed at 1097 cm^− 1^, 1121 cm^− 1^, 1264 cm^− 1^, 1299 cm^− 1^, 1436 cm^− 1^, 1445 cm^− 1^, and 1455 cm^− 1^ corresponding to phospholipid bands in the platelet membrane exhibit a remarkable increased intensity during the active state. In addition, increased intensity of tyrosine and phenylalanine bands were observed in active platelets. Such unusual platelet activation can lead to various adverse effects on human health, particularly in patients with CVDs. One significant consequence is the excessive activation of platelets, which can lead to their adherence to plaque formations in arteries, leading to the *worsening* of CVD. Though an atherosclerotic plaque might not show symptoms, it can cause platelet activation and aggregation. From this perspective, the outcome of the present study on individual live platelets using the optical tweezers micro-Raman spectrometer promises potential applications.

## Data Availability

Data used and/or analyzed during the study is available from the corresponding author upon reasonable request.

## References

[1] Flaumenhaft, R. Platelets: out of shape and misbehaving. *Blood J. Am. Soc. Hematol.* **140**, 2188–2190 (2022). 10.1182/blood.2022018189 36422862

[2] Chang, J. C., Chang, H. H., Lin, C. T. & Lo, S. J. The integrin α6β1 modulation of PI3K and Cdc42 activities induces dynamic filopodium formation in human platelets. *J. Biomed. Sci.* **12**, 881–898 (2005). 16228294 10.1007/s11373-005-9021-2

[3] Nurden, A. T., Nurden, P., Sanchez, M., Andia, I. & Anitua, E. Platelets and wound healing. *Front. Biosci.* **13**, 3532–3548 (2008). 18508453 10.2741/2947

[4] Smith, C. W. Release of α-granule contents during platelet activation. *Platelets* **33**, 491–502 (2022). 34569425 10.1080/09537104.2021.1913576

[5] Michelson, A. D., Barnard, M. R., Krueger, L. A., Valeri, C. R. & Furman, M. I. circulating monocyte-platelet aggregates are a more sensitive marker of in vivo platelet activation than platelet surface P-selectin: studies in baboons, human coronary intervention, and human acute myocardial infarction. *Circulation* **104**, 1533–1537 (2001). 11571248 10.1161/hc3801.095588

[6] Haft, J. I. Role of blood platelets in coronary artery disease. *Am. J. Cardiol.* **43**, 1197–1206 (1979). 35967 10.1016/0002-9149(79)90154-1

[7] Khodadi, E. Platelet function in cardiovascular disease: activation of molecules and activation by molecules. *Cardiovasc. Toxicol.* **20**, 1–10 (2020). 31784932 10.1007/s12012-019-09555-4

[8] Kannan, M., Ahmad, F. & Saxena, R. Platelet activation markers in evaluation of thrombotic risk factors in various clinical settings. *Blood Rev.* **37**, 100583 (2019). 31133440 10.1016/j.blre.2019.05.007

[9] Nash, G., Turner, L., Scully, M. & Kakkar, A. Platelets and cancer. *Lancet Oncol.* **3**, 425–430 (2002). 12142172 10.1016/s1470-2045(02)00789-1

[10] Keji, C., Mei, X. & Huijun, Y. The relationship between platelet activation and coronary heart disease and blood-stasis syndrome. *J. Capital Med. Univ.* **29**, 266 (2008).

[11] Heusch, G., Schulz, R., Baumgart, D., Haude, M. & Erbel, R. Coronary microembolization. *Prog. Cardiovasc. Dis.* **44**, 217–230 (2001). 11727279 10.1053/pcad.2001.26968

[12] Gawaz, M. Role of platelets in coronary thrombosis and reperfusion of ischemic myocardium. *Cardiovascular. Res.* **61**, 498–511 (2004). 10.1016/j.cardiores.2003.11.036 14962480

[13] Ashkin, A., Dziedzic, J. M. & Yamane, T. Optical trapping and manipulation of single cells using infrared laser beams. *Nature* **330**, 769–771 (1987). 3320757 10.1038/330769a0

[14] Ashkin, A. & Dziedzic, J. M. Optical trapping and manipulation of viruses and bacteria. *Science* **235**, 1517–1520 (1987). 3547653 10.1126/science.3547653

[15] Bankapur, A., Zachariah, E., Chidangil, S., Valiathan, M. & Mathur, D. Raman tweezers spectroscopy of live, single red and white blood cells. *PLoS One*. **5**, e10427 (2010). 20454686 10.1371/journal.pone.0010427 PMC2861675

[16] Mithun, N., Lukose, J., Shastry, S., Mohan, G. & Chidangil, S. Human red blood cell behaviour in hydroxyethyl starch: probed by single cell spectroscopy. *RSC Adv.* **10**, 31453–31462 (2020). 35520664 10.1039/d0ra05842d PMC9056550

[17] Lukose, J., Mithun, N., Mohan, G., Shastry, S. & Chidangil, S. Normal saline-induced deoxygenation of red blood cells probed by optical tweezers combined with the micro-raman technique. *RSC Adv.* **9**, 7878–7884 (2019). 35521160 10.1039/c8ra10061f PMC9061285

[18] Lukose, J. et al. Laser Raman tweezer spectroscopy to explore the bisphenol A-induced changes in human erythrocytes. *RSC Adv.* **9**, 15933–15940 (2019). 35521407 10.1039/c9ra01840a PMC9064284

[19] Kato, R., Yano, T. & Tanaka, T. Single-cell infrared vibrational analysis by optical trapping mid-infrared photothermal microscopy. *Analyst* **148**, 1285–1290 (2023). 36811918 10.1039/d2an01917e

[20] Singh, Y., Chowdhury, A., Dasgupta, R. & Majumder, S. K. The effects of lithium on human red blood cells studied using optical spectroscopy and laser trap. *Eur. Biophys. J.* **52**, 91–100 (2023). 36929427 10.1007/s00249-023-01643-2

[21] Shetty, S., Bharati, S., Chidangil, S. & Bankapur, A. Optical trapping and Micro-raman Spectroscopy of Functional Red Blood cells using Vortex Beam for cell membrane studies. *Anal. Chem.* **93**, 5484–5493 (2021). 33764040 10.1021/acs.analchem.0c05204

[22] Bernecker, C. et al. Biomechanics of ex vivo-generated red blood cells investigated by optical tweezers and digital holographic microscopy. *Cells* **10**, 552 (2021). 33806520 10.3390/cells10030552 PMC7998599

[23] Dorta, D. et al. Optical tweezers to measure the elasticity of red blood cells: a tool to study the erythrocyte response to antimalarials. *Front. Malar.* **2**, 1362644 (2024).

[24] Nafie, L. A. Theory of Raman scattering. *Practical Spectrosc. Ser.* **28**, 1–10 (2001).

[25] Blair, T. A., Frelinger, I. I. I. & Michelson, A. A. L. D. in *Platelets* 627–651Elsevier, (2019).

[26] Lin, M. et al. Laser tweezers Raman spectroscopy combined with machine learning for diagnosis of Alzheimer’s disease. *Spectrochim. Acta Part A Mol. Biomol. Spectrosc.* **280**, 121542 (2022). 10.1016/j.saa.2022.121542 35792482

[27] Barkur, S., Bankapur, A., Chidangil, S. & Mathur, D. Effect of infrared light on live blood cells: role of β-carotene. *J. Photochem. Photobiol., B*. **171**, 104–116 (2017). 28495612 10.1016/j.jphotobiol.2017.04.034

[28] Czamara, K. et al. Raman spectroscopy of lipids: a review. *J. Raman Spectrosc.* **46**, 4–20 (2015).

[29] Fox, C. B., Uibel, R. H. & Harris, J. M. Detecting phase transitions in phosphatidylcholine vesicles by Raman microscopy and self-modeling curve resolution. *J. Phys. Chem. B*. **111**, 11428–11436 (2007). 17850068 10.1021/jp0735886

[30] Zyubin, A. et al. Surface-enhanced Raman spectroscopy for antiplatelet therapy effectiveness assessment. *Laser Phys. Lett.* **17**, 045601 (2020).

[31] Giancaspro, J. et al. Structural determination of model phospholipid membranes by Raman spectroscopy: Laboratory experiment. *Biochem. Mol. Biol. Educ.* **50**, 181–192 (2022). 35050536 10.1002/bmb.21603

[32] De Gelder, J., De Gussem, K., Vandenabeele, P. & Moens, L. Reference database of Raman spectra of biological molecules. *J. Raman Spectroscopy: Int. J. Original Work all Aspects Raman Spectrosc. Including High. Order Processes also Brillouin Rayleigh Scattering*. **38**, 1133–1147 (2007).

[33] García-Rubio, D. L. et al. Analysis of platelets in hypertensive and normotensive individuals using Raman and Fourier transform infrared‐attenuated total reflectance spectroscopies. *J. Raman Spectrosc.* **50**, 509–521 (2019).

[34] Wen, S., Hess, D., Kauffman, J., Collins, J. & Lis, L. Raman spectroscopic and X-ray diffraction studies of the effect of temperature and Ca2 + on phosphatidylethanolamine dispersions. *Chem. Phys. Lipids*. **32**, 165–173 (1983). 6850948 10.1016/0009-3084(83)90051-8

[35] Zhu, G., Zhu, X., Fan, Q. & Wan, X. Raman spectra of amino acids and their aqueous solutions. *Spectrochim. Acta Part A Mol. Biomol. Spectrosc.* **78**, 1187–1195 (2011). 10.1016/j.saa.2010.12.079 21242101

[36] Bevers, E. M., Comfurius, P. & Zwaal, R. F. Changes in membrane phospholipid distribution during platelet activation. *Biochim. et Biophys. Acta (BBA)-Biomembranes*. **736**, 57–66 (1983). 10.1016/0005-2736(83)90169-4 6418205

[37] Koseoglu, S. et al. Analytical characterization of the role of phospholipids in platelet adhesion and secretion. *Anal. Chem.* **87**, 413–421 (2015). 25439269 10.1021/ac502293p PMC4287828

[38] Hankins, H. M., Baldridge, R. D., Xu, P. & Graham, T. R. Role of flippases, scramblases and transfer proteins in phosphatidylserine subcellular distribution. *Traffic* **16**, 35–47 (2015). 25284293 10.1111/tra.12233 PMC4275391

[39] Bevers, E. M. et al. Generation of prothrombin-converting activity and the exposure of phosphatidylserine at the outer surface of platelets. *Eur. J. Biochem.* **122**, 429–436 (1982). 7060583 10.1111/j.1432-1033.1982.tb05898.x

[40] Reddy, E. C. & Rand, M. L. Procoagulant phosphatidylserine-exposing platelets in vitro and in vivo. *Front. Cardiovasc. Med.* **7**, 15 (2020). 32195268 10.3389/fcvm.2020.00015 PMC7062866

[41] Ghoshal, K. & Bhattacharyya, M. Overview of platelet physiology: its hemostatic and nonhemostatic role in disease pathogenesis. *Sci. World J.* **2014** (2014). 10.1155/2014/781857 PMC3960550 24729754

[42] Bevers, E. M. & Williamson, P. L. Getting to the outer leaflet: physiology of phosphatidylserine exposure at the plasma membrane. *Physiol. Rev.* **96**, 605–645 (2016). 26936867 10.1152/physrev.00020.2015

[43] Wang, J. et al. The role of phosphatidylserine on the membrane in immunity and blood coagulation. *Biomark. Res.* **10**, 4 (2022). 35033201 10.1186/s40364-021-00346-0 PMC8760663

[44] Shin, E. K., Park, H., Noh, J. Y., Lim, K. M. & Chung, J. H. Platelet shape changes and cytoskeleton dynamics as novel therapeutic targets for anti-thrombotic drugs. *Biomolecules Ther.* **25**, 223 (2017). 10.4062/biomolther.2016.138 PMC5424631 27871158

[45] Patel-Hett, S. et al. Visualization of microtubule growth in living platelets reveals a dynamic marginal band with multiple microtubules. *Blood J. Am. Soc. Hematol.* **111**, 4605–4616 (2008). 10.1182/blood-2007-10-118844 PMC2343595 18230754

[46] Hartwig, J. H. The platelet cytoskeleton. *Platelets* **2**, 75–97 (2013).

[47] Hartwig, J. H. in *Seminars in Hematology.* S94-S100 (Elsevier).

[48] Gross, B. S. et al. Tyrosine phosphorylation of SLP-76 is downstream of syk following stimulation of the collagen receptor in platelets. *J. Biol. Chem.* **274**, 5963–5971 (1999). 10026222 10.1074/jbc.274.9.5963

[49] Crovello, C. S., Furie, B. C. & Furie, B. Histidine phosphorylation of P-selectin upon stimulation of human platelets: a novel pathway for activation-dependent signal transduction. *Cell* **82**, 279–286 (1995). 7543025 10.1016/0092-8674(95)90315-1

[50] Rinder, H. et al. Activation in stored platelet concentrates: correlation between membrane expression of P-selectin, glycoprotein IIb/IIIa, and beta‐thromboglobulin release. *Transfusion* **33**, 25–29 (1993). 7678708 10.1046/j.1537-2995.1993.33193142305.x

[51] De Candia, E. Mechanisms of platelet activation by thrombin: a short history. *Thromb. Res.* **129**, 250–256 (2012). 22137742 10.1016/j.thromres.2011.11.001

[52] Clark, S. R. et al. Characterization of platelet aminophospholipid externalization reveals fatty acids as molecular determinants that regulate coagulation. *Proceedings of the National Academy of Sciences* 110, 5875–5880 (2013). 10.1073/pnas.1222419110 PMC3625294 23530199

[53] Šimák, J., Holada, K., Janota, J. & Straňák, Z. Surface expression of major membrane glycoproteins on resting and TRAP-activated neonatal platelets. *Pediatr. Res.* **46**, 445–445 (1999). 10509366 10.1203/00006450-199910000-00014

[54] Wood, B. R., Hammer, L., Davis, L. & McNaughton, D. Raman microspectroscopy and imaging provides insights into heme aggregation and denaturation within human erythrocytes. *J. Biomed. Opt.* **10**, 014005–014005 (2005). 10.1117/1.1854678 15847586

